# Application of NanoSIMS and isotopic labeling to analyze the spatial distribution of the plant iron chelator nicotianamine

**DOI:** 10.1016/j.jbc.2025.110478

**Published:** 2025-07-11

**Authors:** Tomoko Nozoye, Miyuki Takeuchi, Fabian Hollmann, Stephan Clemens

**Affiliations:** 1 Meiji Gakuin University, Center for Liberal Arts, Yokohama, Japan; 2 The University of Tokyo, Department of Global Agricultural Sciences, Laboratory of Plant Biotechnology, Tokyo, Japan; 3 The University of Tokyo, Graduate School of Agricultural and Life Sciences, Department of Biomaterial Sciences, Tokyo, Japan; 4 University of Bayreuth, Department of Plant Physiology, Bayreuth, Germany

**Keywords:** NanoSIMS, iron, nicotianamine, transport, *chloronerva*

## Abstract

Nicotianamine (NA) is an iron (Fe) chelator responsible for the Fe translocation and transport within the plant body and cells. NA is also hypothesized to be involved in Fe homeostasis as a Fe deficiency signal transducer in plants. This study visualized the distribution of stable isotope-labeled NA in plants by high-resolution secondary ion mass spectrometry (NanoSIMS) to better understand the mechanisms of NA-mediated Fe translocation, transport, and Fe homeostasis. Stable isotope-labeled NA (^15^N-NA) was produced by heterologous expression of an NA synthase in yeast and supplied to wild type (WT) tomato plants and the NA-free mutant *chloronerva* (*chln*). NanoSIMS showed that ^15^N-NA signals were concentrated in tissue surrounding xylem vessels, where Fe accumulated in both WT and *chln*. Our results show that NanoSIMS is a powerful tool for investigating NA localization directly in plants.

The non-proteinogenic amino acid nicotianamine (NA) is an important metal chelator in plants. It is known to bind a range of metal ions, including ferrous iron (Fe^2+^), ferric iron (Fe^3+^), and zinc (Zn^2+^) (1, 2, 3, 4, 5, 6, 7). Fe and Zn belong to the micronutrients essential for practically all living organisms. They contribute to various biological functions as co-factors of proteins but can be deleterious when present in excess or incorrectly distributed. Therefore, a highly regulated and complex homeostatic network maintains metal content within the narrow physiological range between deficiency and toxicity. In addition, in the case of Fe, the redox changes between Fe^2+^ and Fe^3+^ may generate a highly reactive and toxic hydroxyl radical. Both ion forms are of very low solubility at physiological pH values. Fe is therefore always complexed to ligands that increase solubility, allow transport, and decrease redox reactions.

NA was first detected in tobacco leaves (8) but is ubiquitous in the plant kingdom (9) and has been suggested to play an essential role in metal translocation and accumulation in planta. The importance of NA in Fe metabolism was discovered by analysis of the NA-free tomato mutant *chloronerva* (*chln*; (10, 11)). The *chln* mutant has a phenotype indicative of Fe deficiency and is sterile in homozygous genotypes, demonstrating the essential function of NA (12, 13). The simultaneous isolation of the *chln* gene from tomato and of cDNAs encoding NA synthases from barley revealed that NA is synthesized from three molecules of S-adenosylmethionine (SAM) in a one-step reaction catalyzed by NA synthase (NAS; (13, 14, 15)). The *chln* mutation causes a single amino acid exchange in the only tomato NA synthase gene (14). In addition, in *Arabidopsis* and tobacco, NA has been shown to be involved in the translocation not only of Fe, but of Zn and copper (Cu) as well. These findings were derived from analyses of NA-deficient transgenic plants (16, 17). In graminaceous plants, NA is in addition the intermediate of the biosynthesis of the mugineic acid family phytosiderophores (MAs) which are important for the mobilization and uptake of insoluble Fe in the soil (18, 19). Analysis of the transgenic rice plants expressing rice NA synthase 2 (OsNAS2) fused to the green fluorescent protein (GFP) under the control of the OsNAS2 promoter suggested that OsNAS2 localized in vesicular compartments in the cell and moves dynamically along the intracellular transport machinery (20, 21). In addition, it was suggested that this localization and movement may be necessary for the Fe homeostasis functions of NA. Furthermore, since the Fe sensing mechanism in plant cells is largely unknown although several candidate factors have been reported (22), investigating NA localization in planta represents a promising approach in this context as well.

In mammals, NA is reported to inhibit angiotensin-I converting enzyme (ACE), which plays a key role in the control of blood pressure (23). The oral administration of NA displayed ACE inhibitory activity *in vitro* and antihypertensive effects in rats (24). It was shown that intestinal transporters absorb Fe(II) chelated by NA, which contributes to mammalian Fe uptake (25). In addition, NA has been shown to improve spatial learning and memory functions in rats which could contribute to the improvement of Alzheimer's disease (26). The investigation of NA localization is thus important for plant nutrition and relevant for human health.

To date, indirect methods have mainly been used to investigate NA localization in planta, including immunostaining using anti-NA antibodies and ionization time-of-flight mass spectrometry (7, 27, 28). Recent technological advances have greatly enhanced the capability to resolve trace elements in plant tissues and cells spatially. High-resolution secondary ion mass spectrometry (NanoSIMS) uses a focused ion beam to bombard the surface of the materials. The sputtered secondary ions are analyzed in a mass spectrometer, providing elemental information of the sample surface at the subcellular level. Indeed, several elements, from lighter (sulfur (S), phosphorus (P), silicon (Si)) to heavier (arsenic (As), copper (Cu), Fe, Zn), were mapped in the nodes of rice and displayed in sufficient resolution for both subcellular and cellular distribution (29). This study reports an approach to visualize cellular and intracellular localization of NA using the stable nitrogen isotope (^15^N)-labeled NA (^15^N-NA) and NanoSIMS.

## Results

### The production of ^15^N-NA in an *Schizosaccharomyces pombe* strain expressing the *Arabidopsis* NA synthase gene *AtNAS2* fused to a green fluorescent protein (GFP) at its N-terminus (GFP-AtNAS2)

A time course experiment was performed to find the best condition for producing a high concentration of NA (Fig. S1). *S*. *pombe* GFP-AtNAS2 growth reached a plateau after 30 h of cultivation. Growth and the total cell-associated NA contents per culture were positively correlated with Zn concentration in the medium, *i*.*e*. higher in 400 μM Zn compared to normal Zn concentration (13.9 μM). The total cell-associated NA contents per culture were approximately 700 μg/culture in the yeast under 400 μM Zn after 30 h of cultivation. On the other hand, the total cell-associated NA contents per culture under normal Zn conditions (13.9 μM) were approximately 250 μg/culture. The concentrations of NA in the cells were approximately 3000 or 1500 μg/g wet weight under 400 or 13.9 μM Zn conditions, respectively. Between 300 and 500 μM Zn, the growth and NA concentration in the cell did not differ. Both the total cell-associated NA contents per culture and NA concentrations in the cells reached a plateau after 30 h of cultivation as did *S*. *pombe* GFP-AtNAS2 growth regardless of Zn concentrations. Under higher Zn and Fe replete conditions, GFP-AtNAS2 fusion protein was observed as dot-like structures (Fig. S2). This structural fluorescence was not observed under normal Zn concentration in Fe-replete conditions. Since the total NA production (NA content) was higher under high Zn conditions than normal conditions until 30 h of cultivation, this condition (48 h of cultivation in 300 μM Zn) was chosen for further experiments.

### The tracer experiments using ^15^N-NA

**Figure 1:**
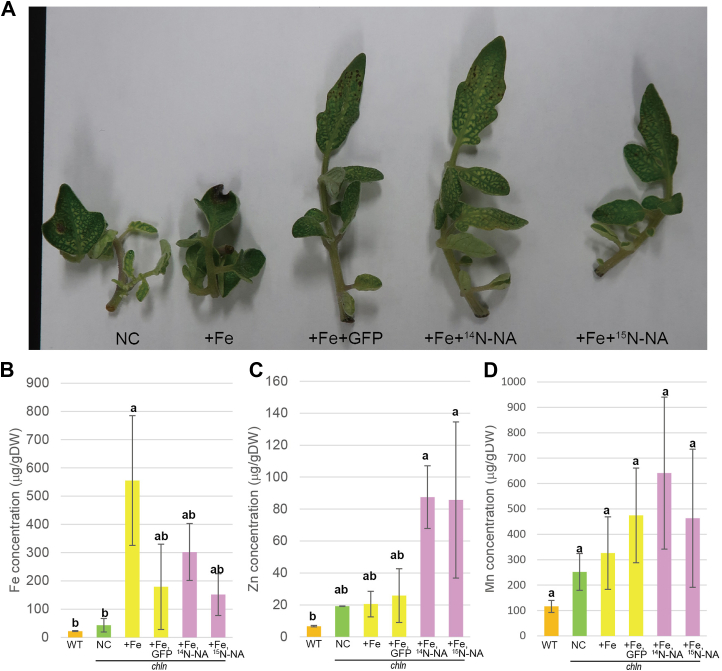
**Tracer experiments with the yeast extracts**. The shoots of *chloronerva* (*chln*) were grown hydroponically and were used for tracer experiments (*A*). The shoots were dipped into the hydroponic culture solution without Fe (negative control; NC), with Fe (+Fe) and the extracts from GFP yeast (+Fe + GFP), Fe and the extracts from GFP-AtNAS2 yeast containing ^14^N-NA (+Fe+^14^N- NA), or Fe and the extracts from GFP-AtNAS2 yeast containing ^15^N-NA (+Fe+^15^N-NA). After 2 days, the Fe (*B*), Zn (*C*), and Mn (*D*) concentrations in shoots were analyzed. The metal concentrations in shoots of wild type (WT) grown hydroponically were also measured. Values represent the means of three biological replicates. *Error bars* represent standard deviations. DW, dry weight. Different letters indicate significant differences according to the Tukey-Kramer HSD test (*n* = 3, *p* < 0.1). The image of *A* is the same as Figure S4*A*. The image of *B* has been derived from the same source as Figure S3*B*.

First, the extracts from GFP-AtNAS2 yeast containing ^14^N-NA or ^15^N-NA, and from GFP yeast containing ^14^N-compounds were supplied to *chln* shoots, respectively, and the concentrations of Fe, Zn, and manganese (Mn) in shoots were analyzed (Fig. 1). Fe was supplied as Fe-EDTA since the use of synthetic Fe-chelates, including Fe-EDTA, has been proven to be a suitable way to provide Fe to plants since the 1950s, and is commonly used in soilless horticulture as well as in high-value, field-grown crops affected by Fe deficiency (30). The metal concentrations in WT shoots were also measured. Fe concentrations did not differ between *chln* and WT. Fe concentration was higher in the *chln* shoots to which the yeast extracts with Fe-EDTA or only Fe-EDTA were added, compared to *chln* shoots dipped into the medium without Fe. The increase in Fe tended to be higher in the shoots that had been treated only with Fe-EDTA compared with shoots to which the yeast extracts with Fe-EDTA were added, although these differences were insignificant. There were no differences in Fe concentrations depending on the presence of NA. Zn concentration in shoots tended to be higher in *chln* compared to WT. Among *chln* shoots, Zn concentrations were higher in the shoots supplied with yeast extracts containing ^14^N-NA and ^15^N-NA than in the others. Mn concentrations did not differ among them.

To check the uptake of N, the existing ratio of ^15^N in the shoots was analyzed (Table S1). The extracts from GFP-AtNAS2 yeast containing ^15^N-NA were supplied to the shoots of *chln* and WT, respectively. The extracts from vector control (VC) yeast, which were grown with a ^15^N-containing medium, were supplied as a negative control. In the shoots of WT and *chln* supplied with the extracts from GFP-AtNAS2 yeast containing ^15^N-NA, ^15^N occupied 1.41 and 2.03 atom%, respectively. This ^15^ N atom% ratio was similar in the shoots of WT and *chln* supplied with the extracts from VC yeast containing ^15^N-compounds: 1.71 and 2.09, respectively. In the non-supplied shoots of WT and *chln*, both ^15^N occupied 0.366 atom%. The total N was approximately 6% in all analyzed samples.

### NanoSIMS imaging

**Figure 2:**
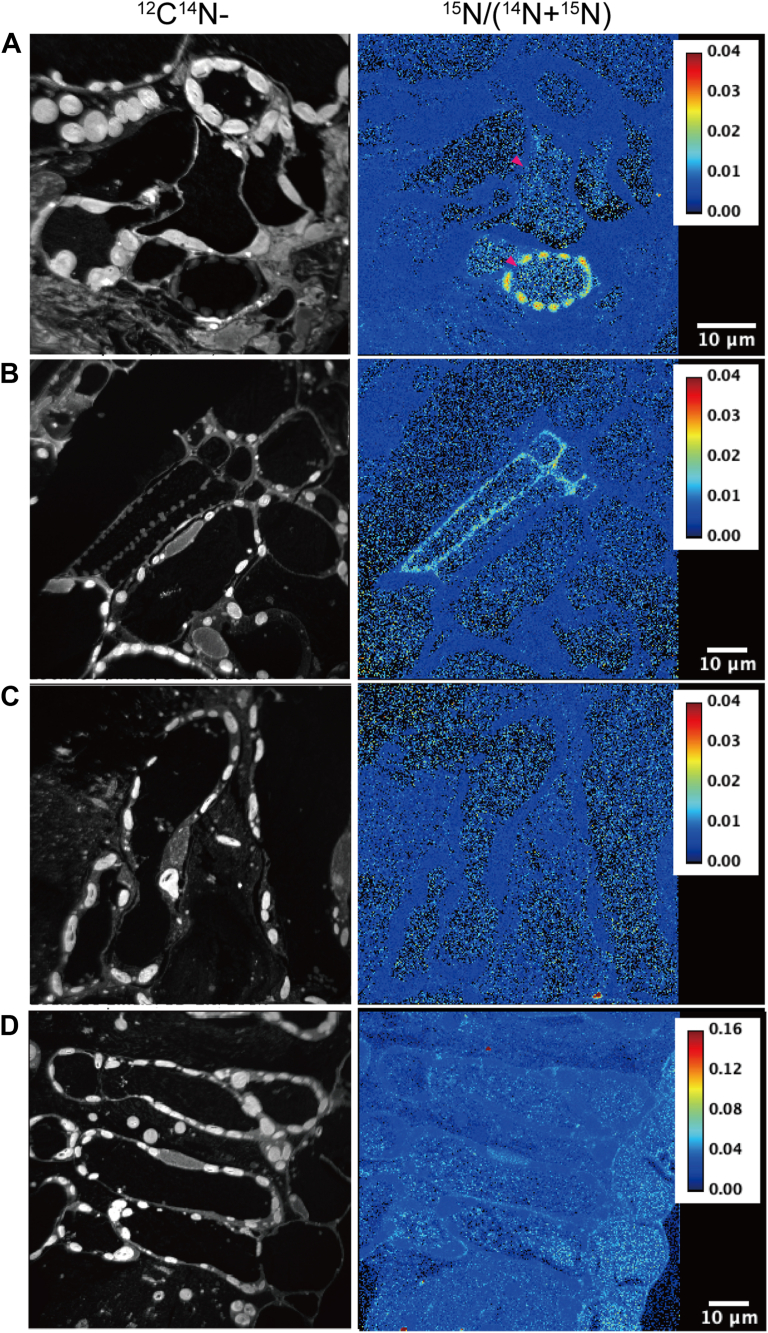
**High-resolution secondary ion mass spectrometry (NanoSIMS) images showing the distribution of ^15^N in the leaves of tomato plants (wild type; A, C, *chloronerva*; B, D)**. After tracer experiments with the extract from GFP-AtNAS2 yeast containing ^15^N-NA (*A*, *B*) and the extracts from the VC yeast grown in the medium containing ^15^N (*C*, *D*), the distribution of ^15^N was analyzed. Bar, 10 μm.

^15^N signals in the leaves of WT and *chln* supplied with the extracts from GFP-AtNAS2 yeast containing ^15^N-NA or the extracts from VC yeast containing ^15^N-compounds were observed (Fig. 2). In the leaves of all analyzed samples, ^15^N signals were observed regardless of the ^15^N-NA existence. However, only in the sample with ^15^N-NA, ^15^N signals were observed to accumulate around the xylem cell walls in both WT and *chln* (Fig. 2, *A* and *B*; Fig. S3, *A* and *B*). The extent of ^15^N accumulation was significantly higher than the natural abundance ratio of ^15^N to ^14^N (Table S1, Fig. S4). Within cells near the xylem, the ^15^N signal in the vacuole tended to be higher than in the cytoplasm. In the mesophyll cells of the sample with ^15^N-NA, ^15^N signals were similar to the natural existing ratio (Fig. S3, *C* and *D*). On the other hand, ^15^N accumulations were observed in the whole cells in both WT and *chln* (Fig. 1, *C* and *D*).

### Fe-staining

**Figure 3:**
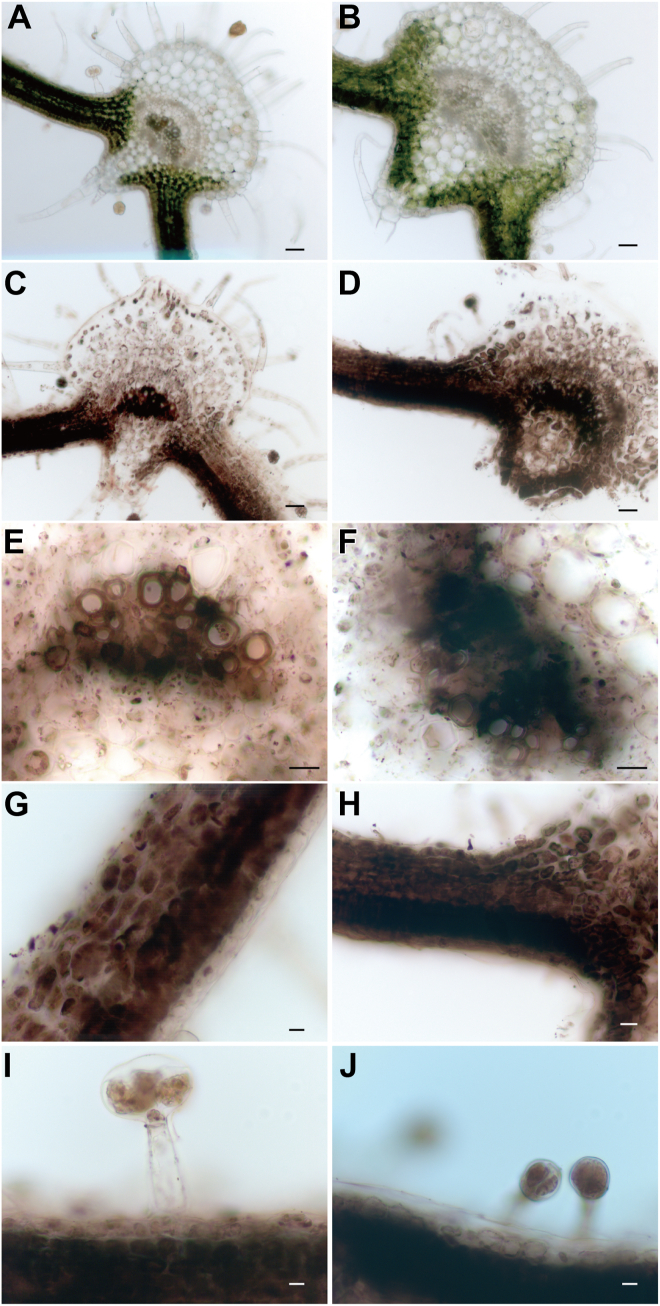
**Localization of Fe by Perl’s Prussian blue-DAB method in the leaves of wild type (WT; C, E, G, I) and *chloronerva* (*chln*; D, F, H, J)**. Cross-sections from leaves before treatments of WT (*A*) or *chln* (*B*) are also represented, respectively. Large vascular bundles (*E*, *F*), the mesophyll cells (*G*, *H*), and the trichome (*I*, *J*) were enlarged. Scale bar, 50 μm (*A*–*D*) or 20 μm (*E*–*J*).

**Figure 4:**
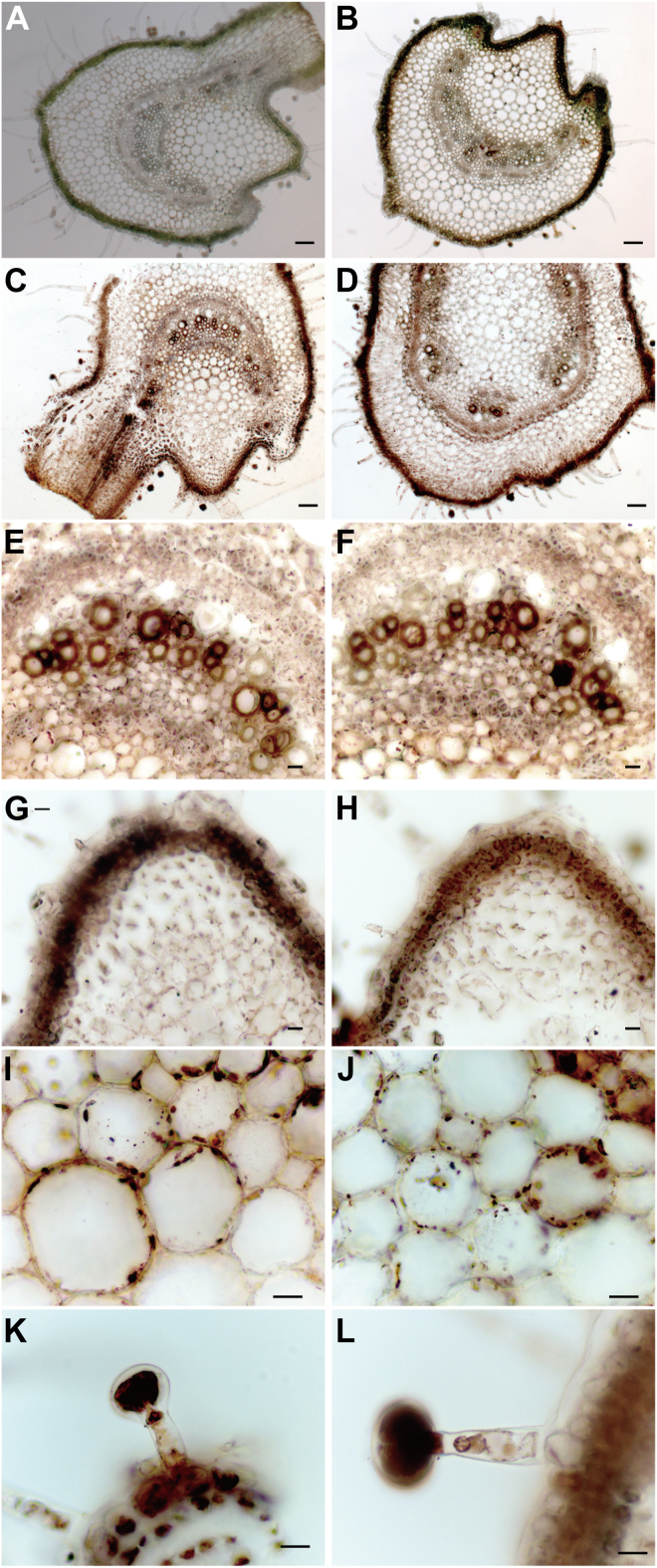
**Localization of Fe by Perl’s Prussian blue-DAB method in the stems of wild type (WT; C, E, G, I, K) and *chloronerva* (*chln*; D, F, H, J, L)**. Cross-sections from stems before treatments of WT (*A*) or *chln* (*B*) were also represented, respectively. Large vascular bundles (*E*, *F*), the epidermis (*G*, *H*), the cortex (*I*, *J*), and the trichome (*K*, *L*) were enlarged. Scale bar, 50 μm (*A*–*D*) or 20 μm (*E*–*L*).

**Figure 5:**
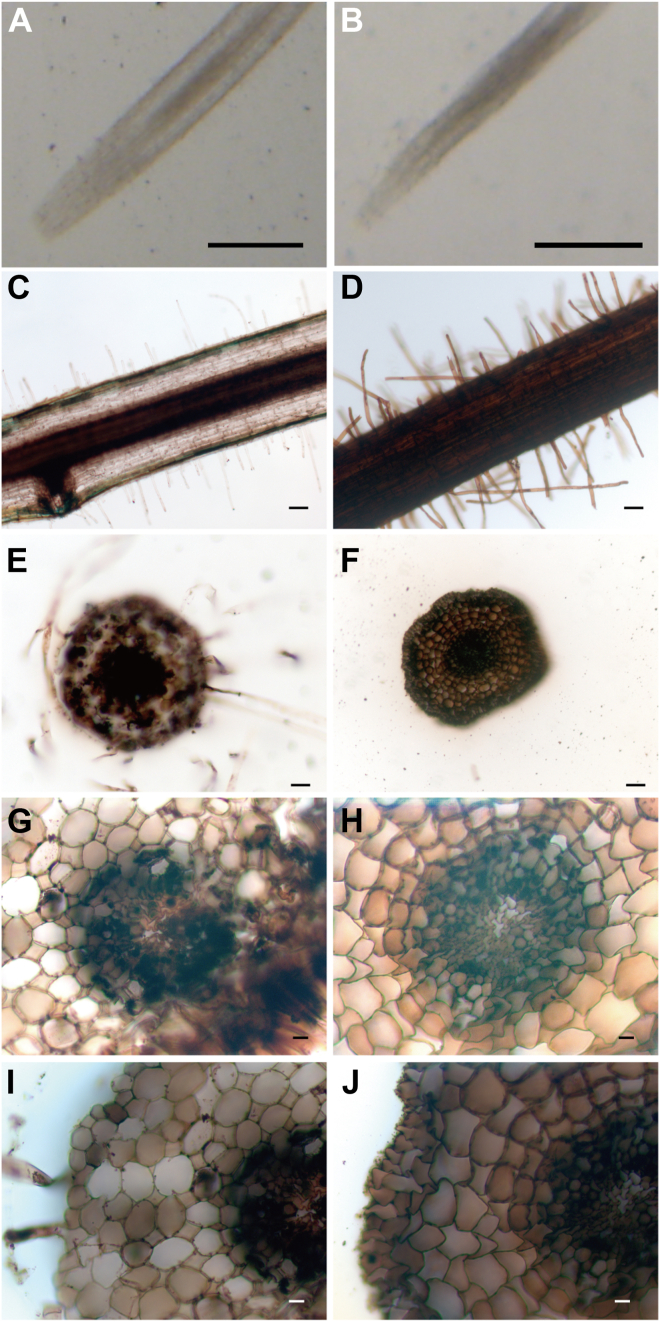
**Localization of Fe by Perl’s Prussian blue-DAB method in the roots of wild type (WT; C, E, G, I) and *chloronerva* (*chln*; D, F, H, J)**. Longitudinal sections from roots before treatments of WT (*A*) or *chln* (*B*) were also represented, respectively. Fe localization was observed in longitudinal (*C*, *D*) and cross (*E*, *F*) sections. The vascular bundles (*G*, *H*) and the cortex (*I*, *J*) were enlarged. Scale bar, 1 mm (*A*, *B*), or 20 μm (*C*–*J*).

Fe was observed in all examined tissues (Figure 3, Figure 4, Figure 5). In leaves, stems, and roots, strong Fe signals were observed in vascular tissue. Fe signals were also detected in the trichomes of the leaves and stems. In the leaves, strong Fe signals were observed in vascular tissue and mesophyll cells (Fig. 3). In *chln*, Fe signals in vascular tissue and palisade cells were higher than in WT. In the mesophyll cells, Fe signals tended to be higher in palisade cells than in spongy parenchyma. Stronger Fe signals in palisade cells were detected in *chln* than in WT. In the stem, Fe was abundant around the xylem and the periphery (Fig. 4). Fe signals surrounding the xylem were especially intense both in WT and in *chln*. These signals did not differ between them. In the cortex cells of the stem, Fe was observed associated with a vesicular structure near the cell membranes. This accumulation pattern did not differ between WT and *chln*. In the roots, Fe accumulation in the vascular bundle was observed in WT (Fig. 5). In contrast, Fe was accumulated in the whole roots, including the root hairs of *chln*. In the epidermis and cortex, Fe was accumulated in the cells, and a dot-like localization as in stems was not observed.

## Discussion

### Zn levels in the medium influenced the NA production and cellular localization of GFP-AtNAS2 in *S*. *Pombe*

NA production in *S*. *pombe* GFP-AtNAS2 increased with culture growth and reached a plateau at 30 h after inoculation (Fig. S1). The yeast cell densities were higher in high Zn conditions than in normal Zn conditions. In addition, total NA contents per culture and the NA concentrations were higher in high Zn conditions, suggesting that high Zn concentrations in the medium promote cell division and NA production. It was reported that the uptake of Zn is tightly regulated in *S*. *pombe* (31). Under high Zn conditions, the growth of *S*. *pombe* was inhibited, while NAS expression rescued a mutant of *S*. *pombe* sensitive to Zn. Consistently, in this study, the growth of *S*. *pombe* GFP-AtNAS2 was higher under high Zn conditions. It confirms that NA production by GFP-AtNAS2 rescues the inhibition of *S*. *pombe* growth under high Zn conditions. It was reported that NA production was detected 6 h after the expression of *AtNAS2*, and accordingly, intracellular Zn concentration increased in *AtNAS2-GFP* expressing *S*. *pombe* (32). The ligands that bind to Zn were reported to be glutathione, histidine, aqueous, and citrate in NA-free *S*. *pombe*. In contrast, half of Zn was bound to NA in AtNAS2-GFP expressing *S*. *pombe*. These results might suggest that the Zn-NA complex can be safely stored in *S*. *pombe* and used properly to promote cell division. The higher NA levels detected under high Zn conditions suggest that, through complex formation with Zn, NA is removed from the equilibrium, which drives further NA synthesis.

We found that GFP-AtNAS2 was localized to vesicular compartments under high Zn and Fe deficient conditions (Fig. S2). This vesicular fluorescence was not observed under normal conditions. GFP only was localized to the cytoplasm and nucleus and the vesicular fluorescence was not observed. This suggests that GFP-AtNAS2 vesicular fluorescence was a feature of this protein exhibited even in a heterologous system. In rice plants, it was reported that OsNAS2-GFP was localized to vesicular compartments and moved dynamically in the cytoplasm (20, 21). However, it was shown that AtNAS2 was localized to the cytoplasm and the vesicular fluorescence was not observed when transiently expressed in tobacco BY-2 cells (21). Recently, it was reported that NA efflux transporters (NAETs) in *A*. *thaliana*, a Nitrate transporter one/peptide transporter family member, localized to secretory vesicles where NA was stored (27). The vesicular compartmentation of NA likely is functionally important for Zn and Fe homeostasis.

### Supplements of 15N-NA and 14N-NA increased Zn concentration in the shoots of chln

The extracts from *GFP-AtNAS2* yeast containing NA tended to increase Zn concentrations in the shoots of *chln* (Fig. 1). These results were consistent with the results that Zn concentrations were increased in the NA-less tobacco leaves treated with a solution containing Fe with NA (16), suggesting that the supplied ^15^N-NA or ^14^N-NA synthesized by GFP-AtNAS2 yeast and supplied by feeding functioned as a metal chelator in the plants. They are also consistent with the role of NA in Zn hyperaccumulation (33, 34). On the other hand, there were no significant differences in Fe concentrations. Since *chln* accumulates high Fe in its leaves while still showing interveinal chlorosis, a typical symptom of Fe deficiency, the tracer experiments of NA have mainly been performed by applying it to the leaves (4, 12, 35, 36, 37). It was shown that an exogenous NA supply to the leaves of the *chln* mutant reduced the high symplasmic Fe concentrations to the level of the WT. It was suggested that NA is important for the unloading of Fe from phloem rather than phloem transport of Fe in *A*. *thaliana* (17) and rice (7). In rice xylem sap, the NA contribution for Fe and Zn translocation was considered low compared to citrate and MAs (38). This might be a reason why Fe concentrations in the shoots of WT and *chln* were not changed by the supply of ^15^N-NA or ^14^N-NA in the GFP-AtNAS2 yeast extracts (Fig. 1).

### NanoSIMS revealed that ^15^N-NA accumulated in cells surrounding the xylem, consistent with Fe localization

Since ^15^N compounds were supplied to the base of the shoots, the high accumulation of ^15^N in the cells around the xylem might suggest that ^15^N-NA was taken up from the xylem and then transported into the surrounding cells (Fig. 2). It was reported that Fe concentrations in the cell wall of roots were extremely low in *chln* relative to WT, and Fe concentrations in the cytoplasm and vacuole were higher in *chln* than in WT (39). When applying NA, these differences between *chln* and WT disappeared, suggesting that NA is necessary to solubilize and utilize the apoplastic Fe. X-ray Absorption Near-edge Structure (XANES) also showed that the presence of ferrous Fe was lower in the *chln* leaf veins than in WT or interveinal areas, suggesting that NA may be involved in maintaining Fe in the ferrous form in the veins of tomato leaves (40). In agreement, Fe localization was mainly observed in the vasculature of the roots, stem, and leaves (Figure 3, Figure 4, Figure 5). Within cells, the ^15^N signal in the vacuole tended to be higher than in the cytoplasm, suggesting that ^15^N-NA might be taken up by the cells and then transported into the vacuole (Fig. 2). Indeed, immunostaining using anti-NA antibodies suggested that NA localized to the cytoplasm under Fe sufficient and deficient conditions and moved to the vacuole under high Fe conditions (41, 42). It was proposed that NA might function as a Fe scavenger to protect cells by sequestration to the vacuole. However, the ^15^N signals in the cytoplasm and vacuole were observed both in the sample with ^15^N-NA and the sample with the extract from VC grown with ^15^N (Fig. 2). Similarly, the concentrations of ^15^N did not differ among the samples in both WT and *chln* (Table S1). In addition, there is a possibility that the observed signal might be generated by an unappreciated NA metabolite. To address this, a metabolomic approach would be required using ^15^N as an isotope tracer. Further adaptation of nanoSIMS to several tissues under several conditions could help determine the role of NA in Fe transport.

In conclusion, this study showed that NanoSIMS combined with the application of isotopic labeling has great potential to identify the movement of molecules such as the metal chelator NA at the cellular and tissue levels. Further improvements could contribute to advancing the physiological understanding of NA.

## Experimental procedures

### The production of 15N-NA

*S*. *pombe* cells expressing the *Arabidopsis* NA synthase gene *AtNAS2* fused to a green fluorescent protein (GFP) at its N-terminus (GFP-AtNAS2) were generated, and NA was synthesized as described previously (32, 43). Briefly, GFP-AtNAS2 was first grown in Edinburgh’s Minimal medium (EMM) and then transferred ^to^ the EMM containing 300 μM ZnSO_4_. Starting at an OD600 of 0.1, a 50-ml culture was grown for 2 days. For the harvest, the culture was centrifuged and washed once with water. The pellets were freeze-dried, and intracellular NA was extracted with water. NA in the extracts was quantified by liquid chromatography/electrospray ionization time-of-flight mass spectrometry (LC/ESI-TOF-MS) as described previously (38, 44). To produce NA labeled with stable nitrogen isotopes (^15^N-NA), 15N labeled NH_4_Cl (15NH_4_Cl) was used instead of NH_4_Cl in the EMM. Yeast extracts containing ^14^N-NA or ^15^N-NA were used for further analysis. As control experiments, a yeast extract of *S*. *pombe* transformed with GFP-pSLF172 cultured with NH_4_Cl (designated as GFP) was used for metal concentration analysis, and an extract of *S*. *pombe* transformed with pSLF172 (vector control: designated as VC) cultured with ^15^NH_4_Cl was used for NA distribution analysis using NanoSIMS.

### Plant growth

Seeds of the *chln* mutant (*Lycopersicon esculentum* Mill. Cv. *chloronerva*) and its wild type (WT, ‘Bonner Beste’) were germinated on soil. After germination, the soil surrounding the roots was washed out and plants were transferred to a hydroponic culture containing a nutrient solution of the following composition: 0.7 mM K_2_SO_4_, 0.1 mM KCl, 0.1 mM KH_2_PO_4_, 2.0 mM Ca(NO_3_)_2_, 0.5 mM MgSO_4_, 10 μM H_3_BO_3_, 0.5 μM MnSO_4_, 0.2 μM CuSO_4_, 0.5 μM ZnSO_4_, 0.05 μM Na_2_MoO_4,_ and 0.1 mM Fe-EDTA.

Plants were cultivated in a greenhouse at 25 °C with 10 h day^-1^ of natural light.

### Tracer experiment

The treatment with NA was done according to the method described previously (16) with some modifications. The shoots of *chln* or WT were cut at the base of the stem and dipped into the hydroponic culture medium containing the yeast extract. For metal concentration analysis, 50 μl of yeast extracts (^14^N-NA or ^15^N-NA) were added to a 3 mL hydroponic culture medium estimated to contain approximately 50 μM NA with a FeSO_4_ concentration of 100 μM. As a control, yeast extract from *GFP*-expressing cells was used. Each solution contained a nutrient solution similar to a hydroponic culture, except Fe-EDTA. Treatment was performed in a greenhouse at 25 °C with 10 h day^-1^ of natural light. After 6 days, the plants were washed with Milli-Q water, dried at 80 °C, and digested with nitric acid.

For NanoSIMS, 100 μl of yeast extracts (^15^N-NA or VC) were added to a 6 ml hydroponic culture medium. After 2 days, following quick freeze-fixation and resin embedding, thin sections were prepared. Briefly, a portion of the leaf was cut and immediately frozen using a high-pressure freezer (BAL-TEC HPM 010; Bal-Tec/Leica, Wetzlar, Germany). The high-pressure frozen tissues were freeze-substituted in 2% osmium tetroxide (OsO_4_) in acetone under the following conditions: −80 °C for 3 days, −20 °C for 1 day, 4 °C overnight, and room temperature for 2 h. The specimens were subsequently washed with acetone and embedded in Spurr’s resin (Polysciences, Warrington, PA). Sections of 0.5 μm thickness were prepared using an ultramicrotome (Leica Ultracut UCT; Leica Microsystems). The sections were placed onto silicon substrates and coated with gold.

### Metal concentration

Fe, Zn, Mn, and Cu concentrations were determined using inductively coupled plasma-mass spectrometry, as described previously (20). The ratio of ^15^N abundance and total N amount were analyzed with ThermoFisher Scientific Flash 2000 -DELTA^plus^ Advantage ConFlo III System by Shoko Science Cooperation.

### NanoSIMS

A NanoSIMS 50L instrument was used to analyze ^15^N distribution with high resolution. One-μm thick sections prepared from resin-embedded plant samples were examined with a 16-keV Cs^+^ ion beam, and negative secondary ions generated from the sample surface were analyzed using a double-focusing mass spectrometer. Images of ^12^C^-^, ^12^C^14^N^-^ and ^12^C^15^N^-^ were obtained, and the NanoSIMS data were analyzed using ImageJ with the OpenMIMS plugin (https://github.com/BWHCNI/OpenMIMS/wiki/Installation).

### Histochemical detection of Fe

For visualization of Fe deposition in planta, Fe staining was performed as described previously with slight modifications (45). Briefly, the leaf, stem, and roots of tomato plants were cut with a scalpel into approximately 1-cm sections, and then embedded into 5% agar, and then cut into 80 to 130 μm sections using a DTK-100 microslicer (Dosaka EM Co. Ltd) as described previously (46). The histochemical detection of Fe was carried out using Perls’ Prussian blue staining with 3,3′- diaminobenzidine (DAB)/H_2_O_2_ intensification. Fe staining was observed using an Axiophoto microscope (Carl Zeiss) following the manufacturer’s instructions.

## Data availability

All data are contained within the manuscript.

## Supporting information

This article contains supporting information.

## Conflict of interest

The authors declare that they have no conflicts of interest with the contents of this article.
