# Supplementary material for: Application of NanoSIMS and isotopic labeling to analyze the spatial distribution of the plant iron chelator nicotianamine
Source: J Biol Chem. 2025 Jul 11;301(8):110478. doi: 10.1016/j.jbc.2025.110478 (PMC12344255; doi:10.1016/j.jbc.2025.110478)
Supplement: Supplementary Data [file mmc1.docx]

| **Table S1.** The existing ratio of ^15^N in the shoots | | |
| --- | --- | --- |
|  | **^15^N (Atom %)** | **T-N ratio (%)** |
| WT | 0.366 | 6.02 |
| chloronerva | 0.366 | 5.18 |
| WT + ^15^N-NA | 1.41 | 6.32 |
| WT + ^15^N-VC | 1.71 | 6.37 |
| chloronerva + ^15^N-NA | 2.03 | 5.98 |
| chloronerva + ^15^N-VC | 2.09 | 6.22 |


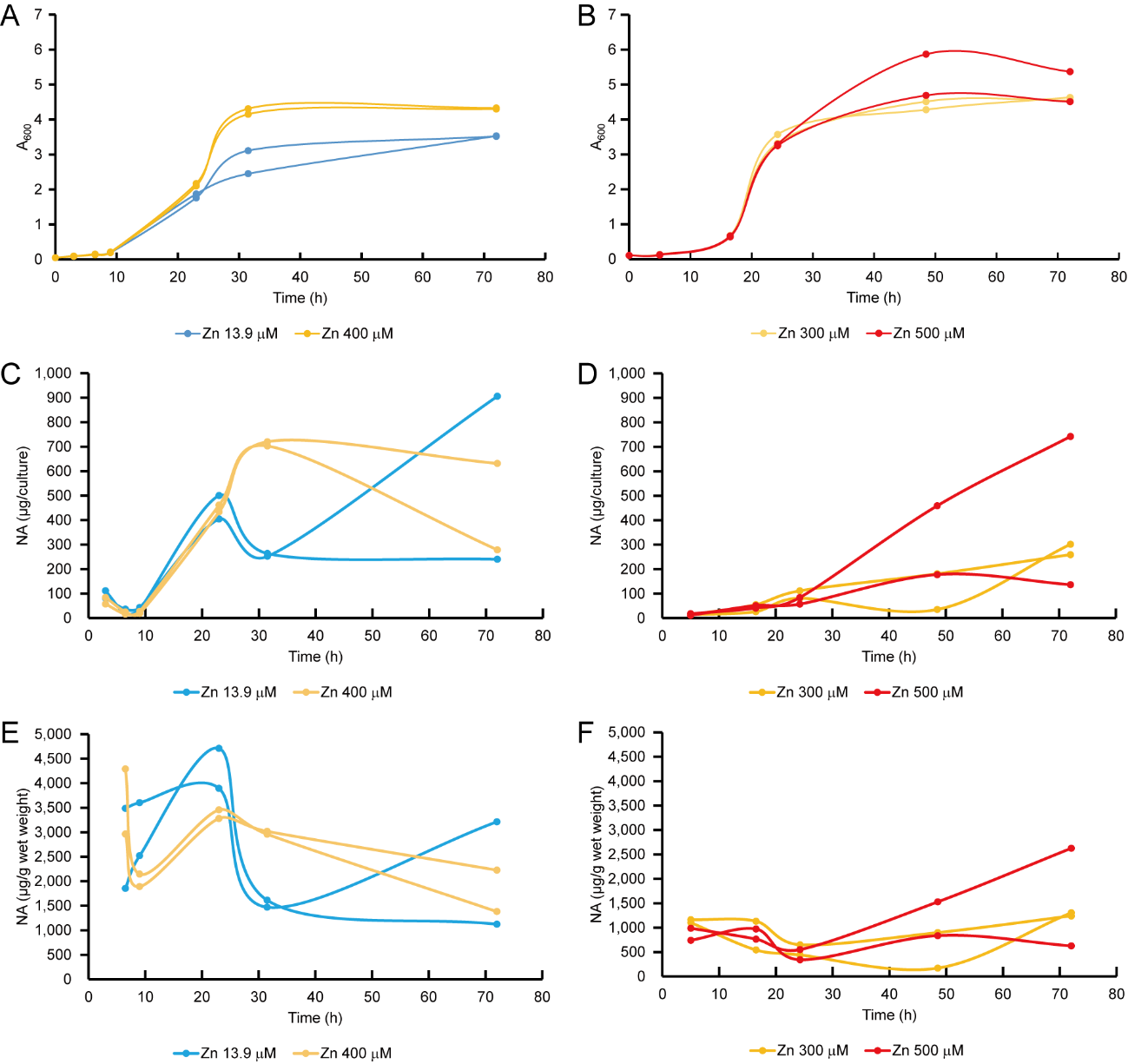


**Figure S1.** Growth condition of *S. pombe* GFP-AtNAS2 to produce ^15^N-NA. Pre-cultured yeast cells were transferred to a new EMM medium with several Zn conditions (13.9, 300, 400, and 500 μM) at time point 0h. The growth curves were determined by measuring the A_600_ of each independent incubation (A, B). Two samples were prepared for each condition. Total NA content (μg/culture; C, D) and the NA concentration (μg/culture; E, F) were determined by LC/ESI-TOF-MS.


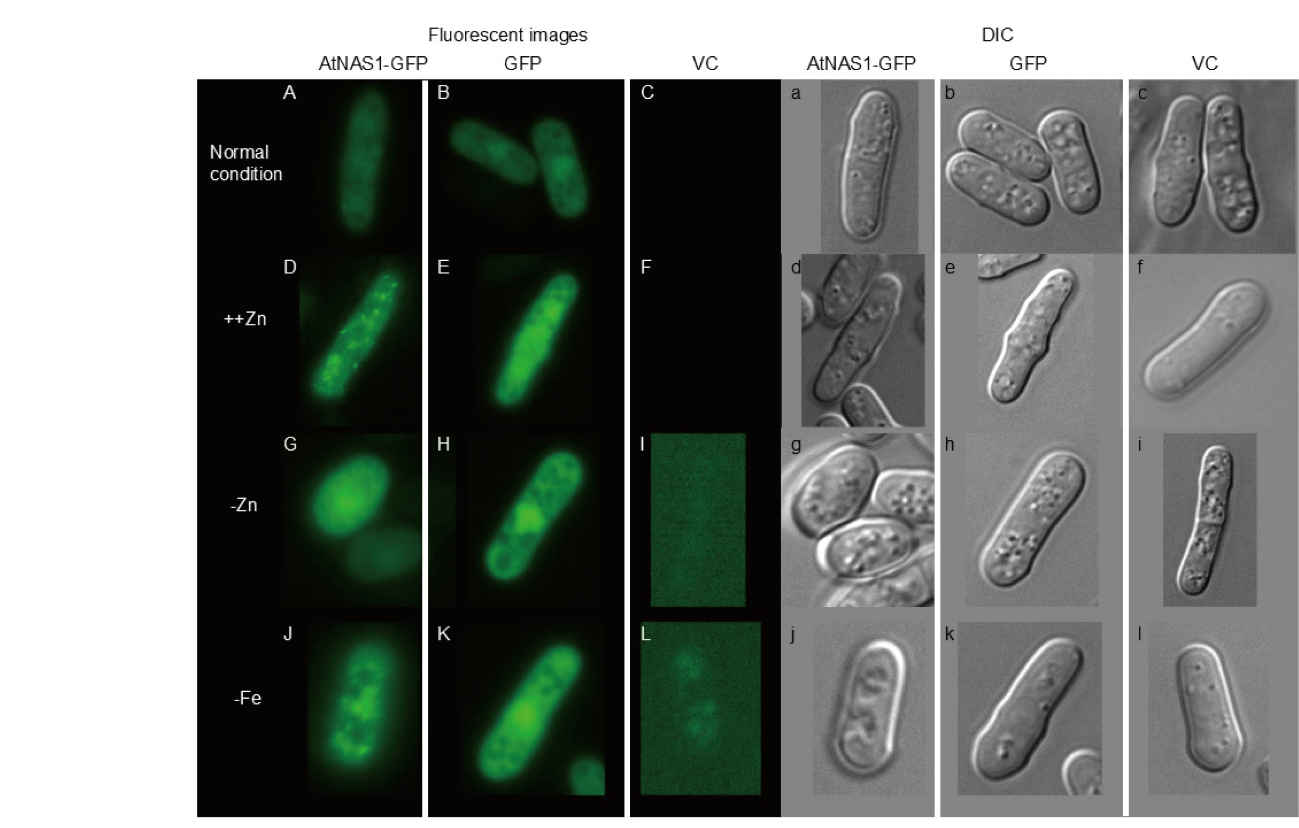


**Figure S2.** Subcellular localization of GFP-AtNAS2 in *S. pombe*. The fluorescence image of GFP (A-L) and differential interference contrast (DIC) image (a-l) were represented. The fluorescent image of the uppercase alphabet corresponds to the DIG image of the lowercase alphabet. *GFP-AtNAS2*, *GFP*, or vector control *S. pombe* were grown under several conditions ( normal Zn (13.9 μM), high Zn (300 μM), without Zn or Fe) and observed by fluorescence microscopy.


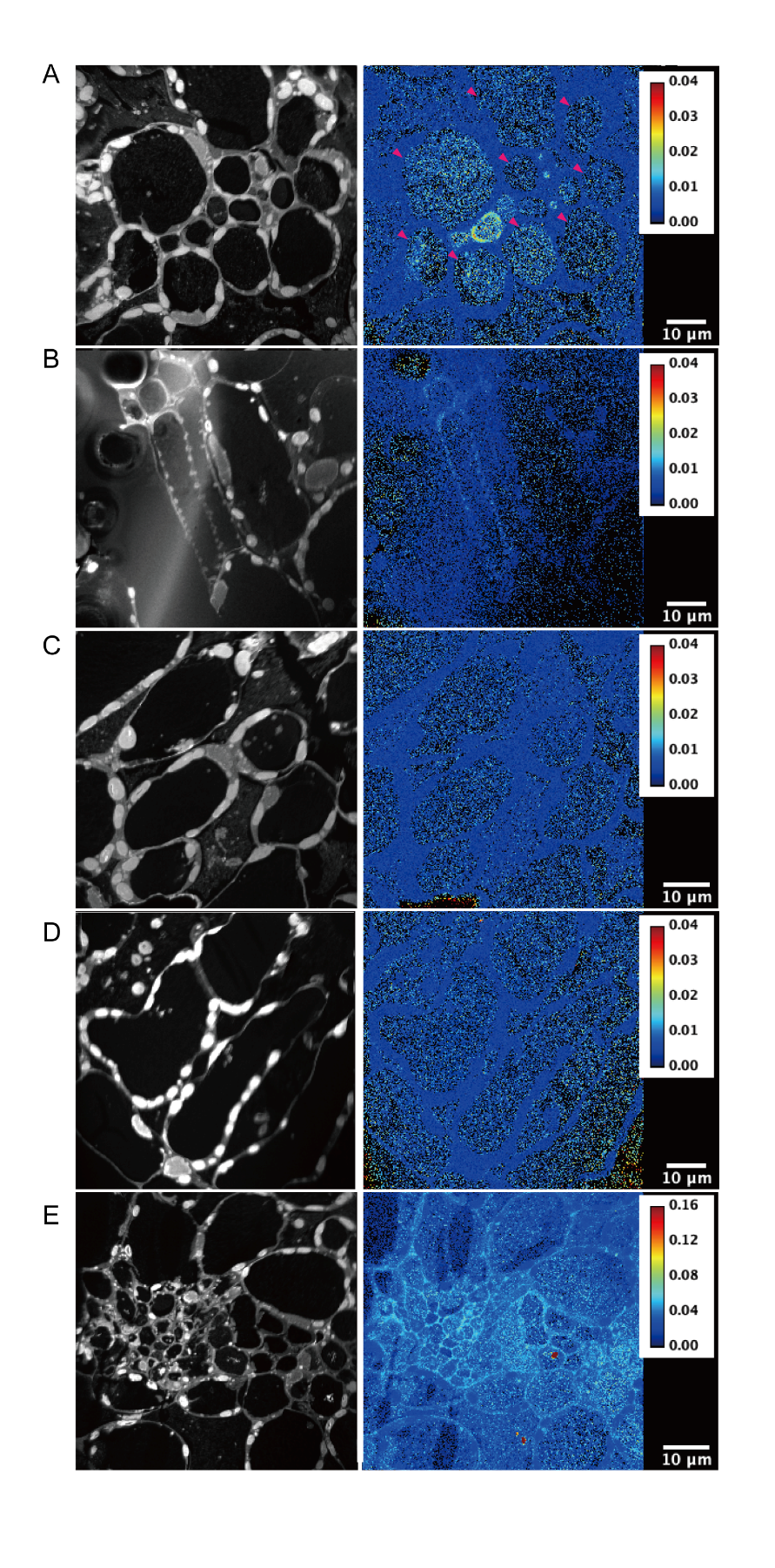


**Figure S3.** High-resolution secondary ion mass spectrometry (NanoSIMS) images showing the distribution of ^15^N in the leaves of tomato plants (wild type; A, C, *chloronerva*; B, D, E). After tracer experiments with the extract from GFP-AtNAS2 yeast containing ^15^N-NA (A-D) and the extracts from the VC yeast grown in the medium containing ^15^N-compoumds (E), the distribution of ^15^N was analyzed. Leaf veins (A, B, E) and mesophyll cells (C, D) were observed. Arrowheads indicate the vacuoles: Bar, 10 μm. The images of B have been derived from the same source as Figure 2B. The images of A, C, and D are the same in the second, third, and fourth panels of Figure S4, respectively.


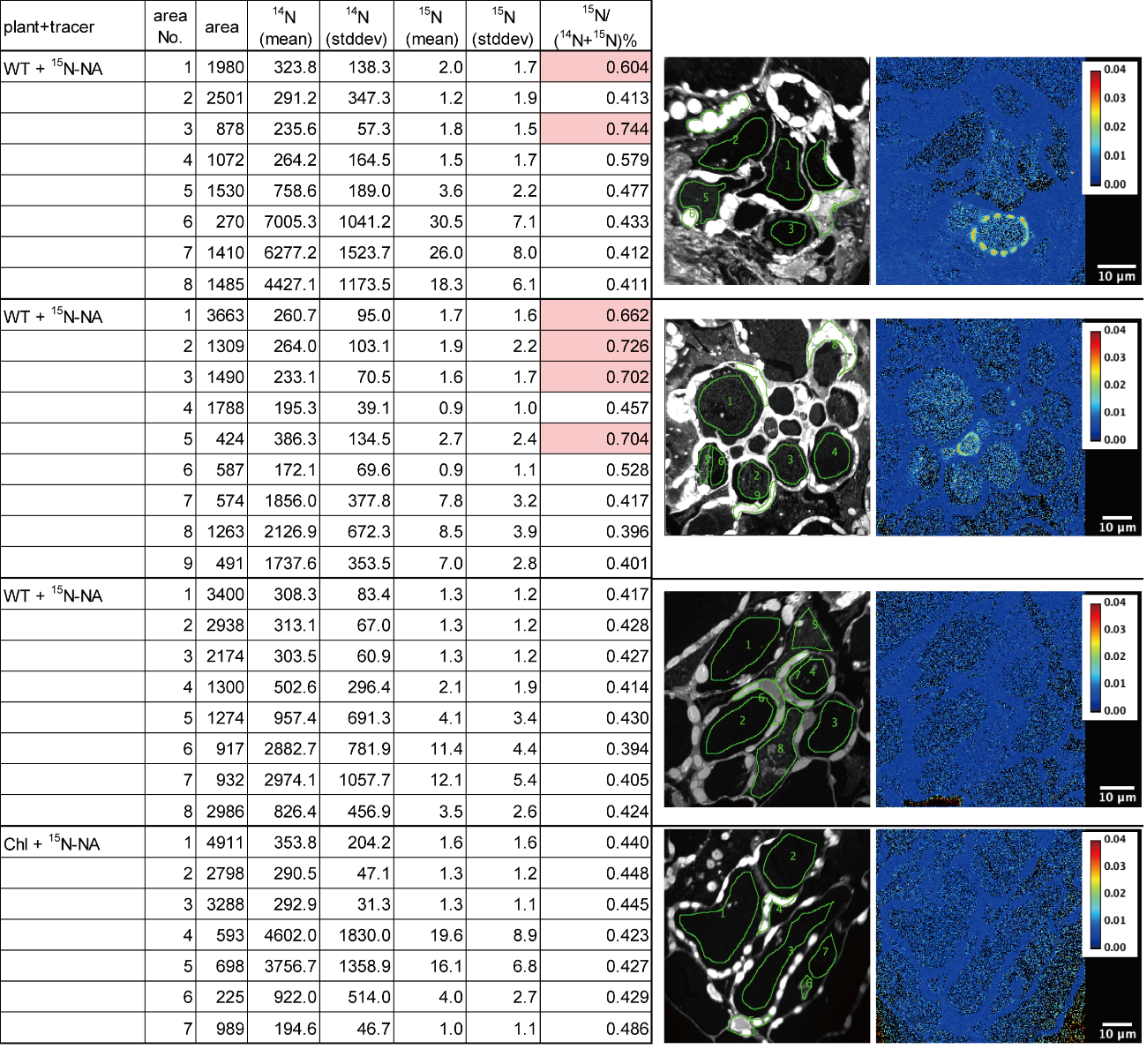


**Figure S4.** Image J software calculated signal intensities of ^14^N and ^15^N in high-resolution secondary ion mass spectrometry (NanoSIMS). The images show the distribution of ^15^N and ^14^N in the leaves of tomato plants (wild type (WT) and *chloronerva* (Chl)) after tracer experiments with the extract from GFP-AtNAS2 yeast containing ^15^N-NA. The images of the first, second, third, and fourth panels are the same as Figure 2A, Figure S3A, S3C, and S3D, respectively. Several areas in each image were captured and the ^15^N existent ratio was calculated by dividing ^15^N signal intensities by ^14^N+^15^N signal intensities. When the ^15^N existent ratio was significantly higher than the natural existent ratio (approximately 0.366 (Table S1), the value column was highlighted with pink color. ^14^N or ^15^N (mean), the average of the signal intensities in the selected area: ^14^N or ^15^N (stddev), the standard division of the signal intensities in the selected area: Bar, 10 μm.
